# Cigarette Smoke Extract Produces Superoxide in Aqueous Media by Reacting with Bicarbonate

**DOI:** 10.3390/toxics9110316

**Published:** 2021-11-22

**Authors:** Jung-Min Park, Haerin Jeong, Yoon-Seok Seo, Van Quan Do, Seong-Jin Choi, Kyuhong Lee, Kyung-Chul Choi, Won Jun Choi, Moo-Yeol Lee

**Affiliations:** 1 BK21 FOUR Team and Integrated Research Institute for Drug Development, College of Pharmacy, Dongguk University, Goyang-si 10326, Gyeonggi-do, Korea; vimrifle@naver.com (J.-M.P.); a26485389@naver.com (H.J.); tjdbstjrtl11@naver.com (Y.-S.S.); dsdoquan@gmail.com (V.Q.D.); mp89@dongguk.edu (W.J.C.); 2 Department of Chemical Assessment, Korea Environment Corporation, Incheon 22689, Korea; choisj@keco.or.kr; 3 Inhalation Toxicology Research Center, Korea Institute of Toxicology, Jeongeup 56212, Jeollabuk-do, Korea; khlee@kitox.re.kr; 4 College of Veterinary Medicine, Chungbuk National University, Cheongju 28644, Chungbuk, Korea; kchoi@cbu.ac.kr

**Keywords:** cigarette smoke, reactive oxygen species, superoxide, bicarbonate

## Abstract

The toxicity of cigarette smoke (CS) is largely attributed to its ability to generate reactive oxygen species (ROS). Reportedly, CS generates superoxide in cell culture systems by stimulating the cells to produce superoxide and through direct chemical reactions with components of the culture media. In this study, we investigated CS-induced superoxide formation in biocompatible aqueous media and its characteristics. Cigarette smoke extract (CSE) and total particulate matter (TPM) were prepared from the mainstream smoke of 3R4F reference cigarettes. CSE and TPM generated superoxide in Hank’s balanced salt solution (HBSS), Dulbecco’s modified Eagle media (DMEM), and blood plasma, but not in distilled water and phosphate-buffered saline. Each constituent of HBSS in solution was tested, and bicarbonate was found to be responsible for the superoxide generation. More than half of the superoxide formation was abolished by pretreating CSE or TPM with peroxidase, indicating that the substrates of peroxidase, presumably peroxides and peroxy acids, mainly contributed to the superoxide production. In conclusion, the presence of bicarbonate in experimental conditions should be considered carefully in studies of the biological activity of CS. Furthermore, the local amount of bicarbonate in exposed tissues may be a determinant of tissue sensitivity to oxidative damage by CS.

## 1. Introduction

Smoking is a well-established risk factor for cardiovascular diseases, cancers, and respiratory disorders. Several studies conducted over the past few decades have uncovered smoking-activated pathogenic events, key aspects of which include cytotoxicity and altered cell growth, inflammation, genetic damage, hypoxia, cardiac and endothelial dysfunction, prothrombotic condition, and abnormal lipid accumulation [1]. Although the detailed mechanisms have not been fully elucidated, the multiple molecular factors of biological systems and a variety of chemicals in cigarette smoke (CS) must be implicated in these processes. This is all the more likely considering the complexity of the signaling pathways leading to these events and the more than 4000 chemical constituents in CS [2]. Among the diverse molecular events induced by smoking, the oxidative stress caused by the excessive or dysregulated production of reactive oxygen species (ROS) is a common factor in a range of toxicological consequences [1,3]. Indeed, CS itself contains free radicals, such as semiquinone, and redox-active compounds, including peroxides, quinones, and catechols [4,5]. CS is also known to stimulate ROS production in cells by activating ROS sources, such as NADPH oxidase and mitochondria [6,7].

CS was observed to generate ROS in in vitro experiments. In a previous study, CS was found to be capable of stimulating cells to produce superoxide by activating NADPH oxidase [8]. In the same experiment, CS generated superoxide even in the absence of cells, indicating superoxide is formed in cell culture media containing CS, regardless of whether there are cells. Similar superoxide formation in aqueous media was reported in another study that tested freshly prepared CS extract [9]; the findings of the study suggested the semiquinones in CS are the culprits behind the superoxide production. However, such superoxide generation is transient due to the high reaction rate constant of semiquinones’ interactions with oxygen in the air [10], and semiquinones are not guaranteed to produce superoxide in aqueous media with a low oxygen concentration [11]. Thus, additional sources and mechanisms seem to be involved in CS-induced superoxide formation in aqueous media [12].

The mechanism of superoxide generation in a cell-free system was investigated with CS preparations. The standard reference cigarette 3R4F was used to generate CS [13,14]. CS is composed of a gas phase and a particulate phase, and both phases were prepared in the form of cigarette smoke extract (CSE) and total particulate matter (TPM), which mainly comprise the gas phase trapped in aqueous solution and the particulate phase trapped in a filter, respectively [15,16]. CS-induced superoxide production was examined in biocompatible aqueous solutions such as cell culture media and buffer solutions. The colorimetric indicator WST-1 and electron paramagnetic resonance (EPR) spectroscopy were employed to detect and identify superoxide. An effort was also made to delineate the constituents of CS and aqueous solutions that are involved in superoxide production.

## 2. Materials and Methods

### 2.1. Reagents

3R4F reference cigarettes (9.4 mg tar and 0.73 mg nicotine) were purchased from the Center for Tobacco Reference Products of the University of Kentucky (Lexington, KY, USA). Fetal bovine serum (FBS), Hank’s balanced salt solution (HBSS), and phosphate-buffered saline (PBS) were obtained from Thermo Fisher Scientific (Waltham, MA, USA). The following chemicals were purchased from Sigma-Aldrich (St. Louis, MO, USA): Dulbecco’s modified Eagle’s medium (DMEM, Cat. No. D2902), 4-hydroxy-2,2,6,6-tetramethylpiperidine-1-oxyl (TEMPOL), superoxide dismutase (SOD), horseradish peroxidase (HRP), diethylenetriaminepentaacetic acid (DTPA), xanthine, xanthine oxidase, catalase, D-mannitol, *meta*-chloroperoxybenzoic acid (*m*CPBA), and ferrous sulfate (FeSO_4_). Other chemicals and sources were as follows: coumarin boronic acid (CBA) (Cayman Chemical, Ann Arbor, MI, USA); 5-(diisopropoxyphosphoryl)-5-methyl-1-pyrroline-*N*-oxide (DIPPMPO) (Enzo Life Sciences, Farmingdale, NY, USA); 2-(4-iodophenyl)-3-(4-nitro-phenyl)-5-(2,4-disulfophenyl)-2H-tetrazolium (WST-1) (Biomax, Seoul, Korea); hydrogen peroxide (H_2_O_2_) (Samchun Chemical, Pyeongtaek, Korea); InstantBlue Coomassie protein stain (Abcam, Cambridge, MA, USA). All other chemicals used were of the highest purity available and were obtained from standard suppliers.

### 2.2. Animals

Male Sprague-Dawley rats at 5 to 6 weeks of age were purchased from Daehan Biolink (Eumseong, Korea). The rats were housed in the laboratory animal facility and acclimated for more than a week before the experiments. The laboratory animal facility was maintained at a controlled temperature of 22 ± 3 °C and relative humidity of 50 ± 20% with ambient noise of less than 60 dB and a 12-h light–dark cycle. Food and water were provided ad libitum.

### 2.3. Preparation of Blood Plasma

Blood plasma was prepared from 7-week-old Sprague-Dawley rats. The rats were anesthetized with ether, and blood was collected from the abdominal aorta using acid-citrate-dextrose (85 mM trisodium citrate, 66.6 mM citric acid, and 111 mM glucose) as an anticoagulant (acid-citrate-dextrose:blood, 1:6). After centrifugation of the blood at 250× *g* for 15 min, the plasma was obtained from the supernatant and used immediately.

### 2.4. Preparation of CSE and TPM

CSE and TPM were prepared as described in our previous study with minor modifications [8]. In accordance with ISO 3402 standards, the cigarettes were conditioned at 22 ± 1 °C and 60 ± 2% relative humidity for 48 h before use. CS was generated by a CSM 2080 30-port smoking machine (CH Technologies, Westwood, NJ, USA) in conformity with ISO standard 3308 regimen: 35 mL puff volume, 2 s puff duration, 60 s between puffs, and no vent blocking. The TPM in mainstream smoke from 30 cigarettes was collected on a 44-mm Cambridge filter pad (GE Healthcare, Little Chalfont, UK). CSE was prepared by passing the remaining smoke through an impinger containing 30 mL PBS for 5 min. TPM trapped on the filter was eluted with dimethyl sulfoxide for 30 min with shaking to make up to 20 mg/mL, and the resulting solution was filtered through a 0.45-µm polytetrafluorethylene filter (Merck Millipore, Darmstadt, Germany). CSE and TPM were immediately dispensed and kept at −80 °C until use. As a standard constituent, nicotine was analyzed with a 6410B triple quadrupole liquid chromatography–mass spectrometer (LC-MS) (Agilent Technologies, Santa Clara, CA, USA). The nicotine concentrations were 24.3 µg/mL and 811.2 µg/mL for CSE and TPM, respectively.

### 2.5. Cells and Cell Culture

Vascular smooth muscle cells (VSMC) were isolated from rat thoracic aortas by enzymatic digestion as previously described [8]. Briefly, aortas were excised and cleaned of connective tissue, fat, and endothelium. Then, the aortas were digested with collagenase and elastase to remove the adventitia and dissociate the cells. Individual cells were plated onto a culture dish and grown in DMEM supplemented with 10% fetal bovine serum, 100 U/mL penicillin, and 100 μg/mL streptomycin. The human alveolar adenocarcinoma cell line A549 was purchased from American Type Culture Collection (Manassas, VA, USA), and grown in the same media as the VSMC. The cells were maintained in a humidified chamber at 37 °C with 5% CO_2_ and subcultured when they reached 80–90% confluence.

### 2.6. Superoxide Measurement Using WST-1

Superoxide was measured with a WST-1 colorimetric probe. CSE or TPM were added to aqueous solutions containing 500 µM WST-1, which included distilled water (DW), PBS (154.00 mM NaCl, 5.60 mM Na_2_HPO_4_, and 1.06 mM KH_2_PO_4_), HBSS (136.98 mM NaCl, 5.37 mM KCl, 1.26 mM CaCl_2_, 0.81 mM MgSO_4_, 0.44 mM KH_2_PO_4_, 0.34 mM Na_2_HPO_4_, 4.16 mM NaHCO_3_, and 5.55 mM glucose), DMEM, DMEM with 10% FBS, and rat blood plasma. For measurement in cultured cells, VSMC or A549 cells were seeded into 96-well plates at a density of 2 × 10^4^ cells/well and grown for 24 h. Cells were treated with 10% CSE in the presence or absence of 10 U/mL SOD. The absorbance at 450 nm was measured for 60 min for CSE or for 30 min for TPM at 37 °C using a SpectraMax M3 microplate reader (Molecular Devices, Sunnyvale, CA, USA). To test the involvement of the HRP substrate in superoxide generation, CSE or TPM was preincubated with HRP for 30 min at 37 °C before superoxide measurement.

### 2.7. EPR Spectroscopy

EPR spin-trapping experiments were performed as in a previous study [17]. CSE was added to a 44 mM NaHCO_3_ solution containing 100 mM DIPPMPO and 100 µM DTPA. The solution was incubated at 37 °C for 30 min in the presence or absence of 100 U/mL SOD, 500 U/mL catalase, 1 mM mannitol, or 50 mM *m*CPBA. Prepared samples were subsequently transferred into a 50-µL capillary tube, and spectra were examined with an EMXplus spectrometer (Bruker, Billerica, MA, USA) working in X-band. The instrumental parameters were as follows: modulation frequency, 100 kHz; modulation amplitude, 1.0 G; receiver gain, 30 dB; microwave power, 9.4 mW; center field, 3435 G; conversion time, 20 ms; time constant, 1.28 ms; sweep time, 30 s. The presented spectra are the averages of five scans. To obtain the typical spectrum for the superoxide or hydroxyl radical, samples were prepared by treating the 44 mM NaHCO_3_ solution with 500 µM xanthine and 10 mU/mL xanthine oxidase or 1 mM FeSO_4_ and 10 mM H_2_O_2_.

### 2.8. Assessment of Protein Carbonylation by CSE

Human albumin was added to 100 µM DTPA-containing DMEM; DMEM with 100 U/mL SOD, 10 mM TEMPOL, or 500 U/mL catalase; or DMEM without NaHCO_3_ to a concentration of 1 mg/mL. These albumin solutions were treated with 10% CSE, and after a 5-min incubation, the reaction was terminated by adding 5 mM of TEMPOL. Carbonylation of the albumin was assessed using the OxyBlot protein oxidation detection kit (Merck Millipore) following the manufacturer’s instructions. Briefly, each sample was subjected to sodium dodecyl sulfate-polyacrylamide gel electrophoresis and transferred to a polyvinylidene difluoride membrane. The carbonylated albumin was probed with a primary antibody specific for 2,4-dinitrophenylhydrazone and a horseradish peroxidase-conjugated secondary antibody, and then visualized with Immobilon Western chemiluminescent HRP substrate (Merck Millipore). Chemiluminescence images were obtained and analyzed with a ChemiDoc XRS+ system equipped with Image Lab software (Bio-Rad laboratories, Hercules, CA, USA). To confirm the amount of loaded albumin, the gels were additionally stained with InstantBlue Coomassie protein stain (Abcam).

### 2.9. Statistical Analyses

The means ± standard errors were calculated for all experimental groups. The data were subjected to one-way analysis of variance followed by Dunnett’s test to determine the significance of the differences relative to the controls, except for the data for albumin carbonylation, which was subjected to two-way analysis of variance followed by Tukey’s honest significant difference test. All statistical analyses were performed using SigmaPlot software ver. 13 (Systat Software, San Jose, CA, USA). A *p*-value less than 0.05 was considered statistically significant.

## 3. Results

### 3.1. CSE Produces Superoxide in Aqueous Solutions

**Figure 1:**
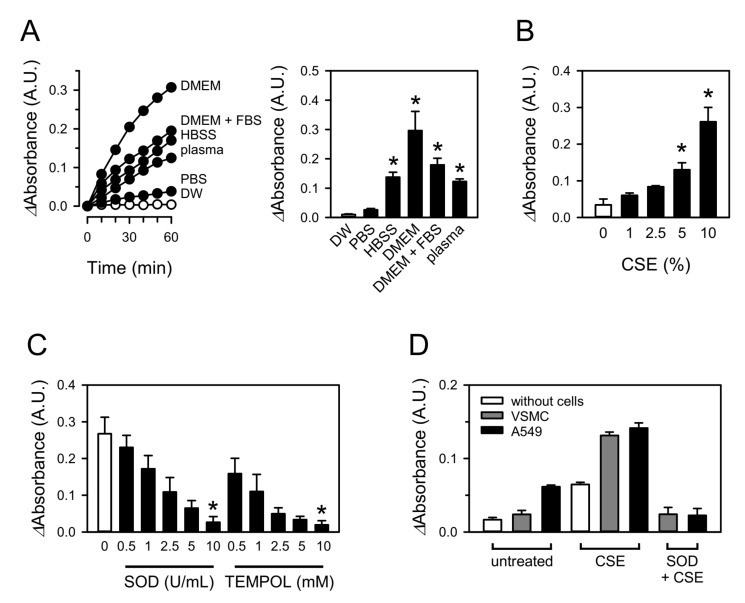
CSE generates superoxide in aqueous solutions. (**A**) CSE was added to aqueous media, DW, PBS, HBSS, DMEM, DMEM containing 10% FBS, or blood plasma, to a 10% concentration. Superoxide formation was measured with WST-1. Representative tracings and the superoxide amounts are presented in the left and right panels, respectively. (**B**) CSE-induced superoxide formation was assessed in DMEM. (**C**) Absorbance was measured in 10% CSE-treated DMEM containing 0.5–10 U/mL SOD or 0.5–10 mM TEMPOL. (**D**) CSE-induced superoxide formation was measured in the presence or absence of VSMC or A549 cells. Values are means ± standard errors (*n* = 9 for A, *n* = 3 for (**B**,**C**), and *n* = 3–6 for (**D**). * *p* < 0.05 vs. corresponding control.

CSE was tested for its ability to produce superoxide in various aqueous media. CSE was used to treat DW, PBS, HBSS, DMEM, DMEM containing FBS, and blood plasma at a concentration of 10%, and superoxide formation was measured using a WST-1 superoxide probe [18]. Adding CSE produced significant amounts of superoxide in HBSS, DMEM with and without FBS, and blood plasma, whereas superoxide generation was minimal in DW and PBS (Figure 1A). Superoxide formation continued for more than 60 min under these experimental conditions. The amount of superoxide generated was greatest in DMEM followed by, in descending order, DMEM containing FBS, HBSS, and blood plasma (Figure 1A). Superoxide production was proportional to the concentration of CSE up to at least 10% in DMEM (Figure 1B). The impact of SOD and the superoxide scavenger TEMPOL on the WST-1 signal was tested in HBSS to confirm the species of reactive oxygen detected with WST-1. As expected, both the SOD and TEMPOL concentration dependently attenuated the CSE-increased WST-1 signal in ranges of 0.5−10 U/mL and 0.5−10 mM, respectively (Figure 1C).

In addition, superoxide generation was assessed in the presence of cells to compare the relative amount of superoxide formed in media and cells. The amount of superoxide generated in the presence of VSMC or A549 was approximately two fold greater than that in the absence of cells (Figure 1D). These values may vary depending on experimental conditions such as the type of cells, number of cells tested, and the concentration of CSE. However, it is clearly noticeable that superoxide from the direct interaction between the media and CSE was quantitatively significant, and it was not negligible compared with that from CSE-stimulated cells.

### 3.2. Bicarbonate in Aqueous Media Is Involved in CSE-Induced Superoxide Production

**Figure 2:**
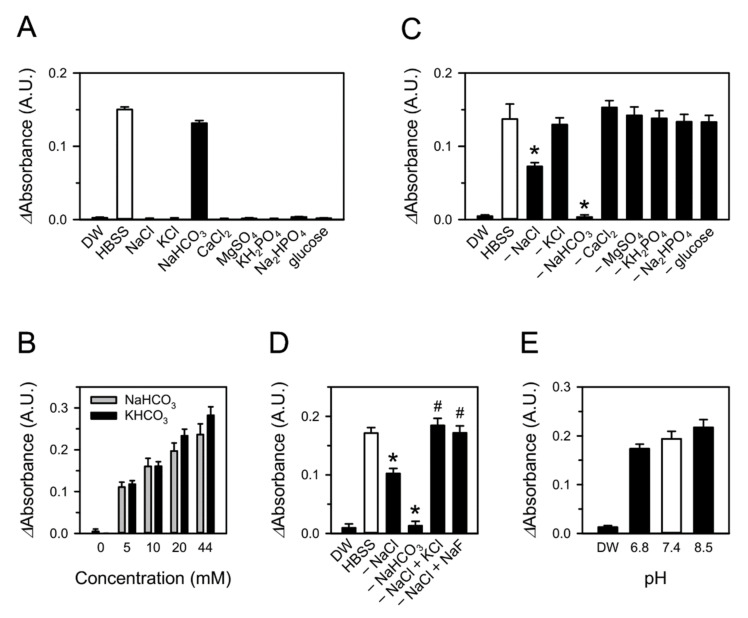
Bicarbonate in aqueous media is involved in CSE-induced superoxide formation. (**A**) Superoxide formation was measured with WST-1 in 10% CSE-treated HBSS or aqueous solutions containing one of each constituent of HBSS. (**B**–**E**) Superoxide formation by CSE was assessed in NaHCO_3_ or KHCO_3_ solution (**B**); HBSS or HBSS lacking one of its constituents (**C**); HBSS, HBSS deficient in NaCl or NaHCO_3_, or HBSS in which NaCl was replaced with NaF or KCl (**D**); and HBSS with a pH adjusted to 8.5 or 6.8 with NaOH or HCl (**E**), respectively. Values are means ± standard errors (*n* = 3). * *p* < 0.05 vs. corresponding control. ^#^ *p* < 0.05 vs. HBSS deficient in NaCl.

Each constituent of the aqueous solutions was examined for its involvement in CSE-induced superoxide production to identify the culprit(s) responsible for superoxide formation. HBSS consists of only eight constituents with the simplest chemical composition among the tested media in which CSE significantly produced superoxide. Accordingly, superoxide formation was tested in eight solutions: NaCl, KCl, NaHCO_3_, CaCl_2_, KH_2_PO_4_, Na_2_HPO_4_, MgSO_4_, and glucose. The concentrations of each constituent were equal to those in HBSS. CSE was added to each solution but superoxide was only produced in the NaHCO_3_ solution, and the amount of superoxide formed was comparable to that produced in HBSS (Figure 2A). Moreover, superoxide production increased in proportion to the NaHCO_3_ concentration up to at least 44 mM, which is the concentration of NaHCO_3_ in DMEM (Figure 2B). A relatively similar amount of superoxide production was observed in KHCO_3_ solution, indicating that bicarbonate (hydrogen carbonate, HCO_3_^−^), rather than sodium ions, are responsible for superoxide generation by CSE.

To confirm the contribution of NaHCO_3_ to the reaction, we tested the amount of superoxide generated in HBSS solutions, each lacking a single constituent. Consistent with previous results, the superoxide generation by CSE was abolished by removing NaHCO_3_ from HBSS (Figure 2C). Unexpectedly, the removal of NaCl from HBSS substantially attenuated superoxide production (Figure 2C,D). This attenuation was fully rescued by supplementing the NaCl-deficient HBSS with an equivalent amount of NaF or KCl (Figure 2D), suggesting that the concentration, rather than the species, of the ions affected superoxide production.

The pH of the bicarbonate solution and HBSS lacking bicarbonate was measured to be approximately 8.5 and 6.8, respectively. CSE-induced superoxide formation was examined in HBSS with pH adjusted to 8.5 or 6.8 with NaOH or HCl, respectively. Superoxide generation appeared to be generally dependent on pH, but it was not dramatically or statistically different (Figure 2E). Differences in the pH of the solutions did not seem to be critical enough to affect the results of previous experiments.

### 3.3. Peroxides in CSE Contribute to Superoxide Production by Reacting with Bicarbonate

**Figure 3:**
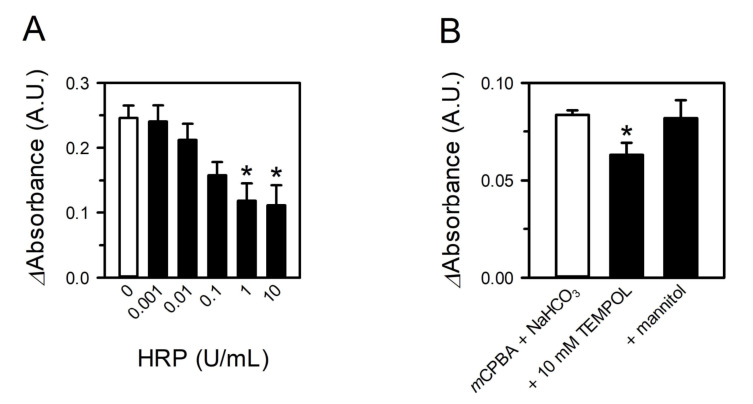
Peroxides in CSE contribute to superoxide production by reacting with bicarbonate. (**A**) CSE was preincubated with HRP and added to 44 mM NaHCO_3_ solution to 10%. Superoxide was measured with WST-1. (**B**) Superoxide formation was assessed with WST-1 for solutions containing 44 mM NaHCO_3_ and 10 mM *m*CPBA. Values are means ± standard errors (*n* = 3 for A and *n* = 4 for B). * *p* < 0.05 vs. corresponding control.

**Figure 4:**
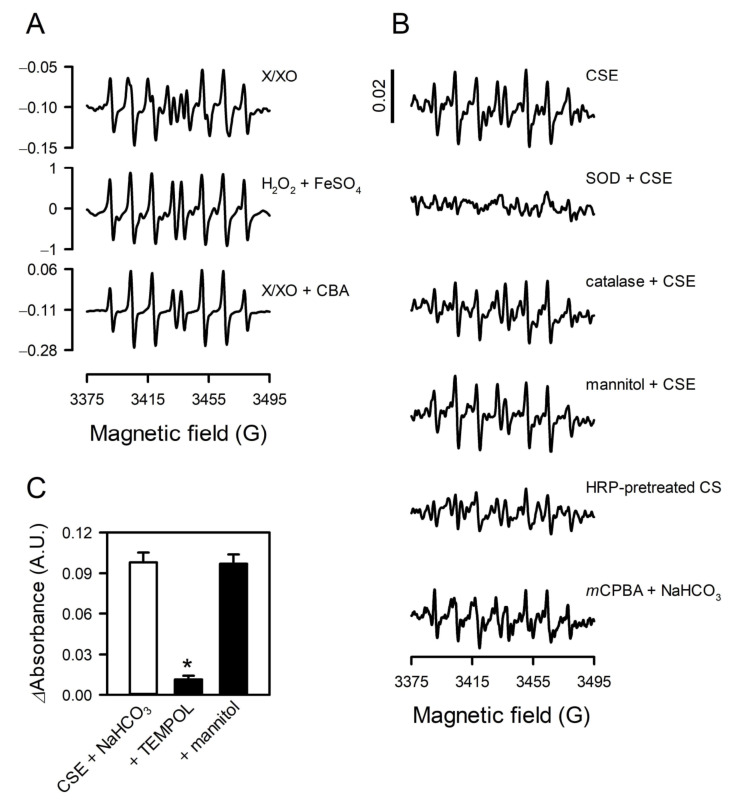
The species of reactive oxygen was reconfirmed to be superoxide. The species of reactive oxygen was identified with a spin trap DIPPMPO. (**A**) Typical EPR spectra for superoxide spin adduct and hydroxyl radical spin adduct were obtained from the specific reaction systems generating superoxide [xanthine (X) + xanthine oxidase (XO)] and hydroxyl radical (H_2_O_2_ + FeSO_4_). (**B**) EPR signals were acquired from 10% CSE-treated NaHCO_3_ solution in the presence or absence of SOD, catalase, or mannitol, or from NaHCO_3_ solution containing HRP-pretreated CSE or *m*CPBA. The spectra shown are the averages of five scans. (**C**) Superoxide production in CSE-treated NaHCO_3_ solution was assessed with WST-1 in the presence of TEMPOL and mannitol. * *p* < 0.05 vs. CSE + NaHCO_3_.

Considering that superoxide can be generated by the chemical reactions of bicarbonate with peroxides [19,20], the peroxides in CSE were postulated to be involved in superoxide production. To test this, CSE was pretreated with HRP to remove peroxides, and then superoxide production was examined in NaHCO_3_ solution. Pretreatment with HRP significantly reduced the capability of CSE to generate superoxide, and the extent of this reduction was dependent on the HRP concentration, with the maximal level of inhibition being about 55% (Figure 3A). Additionally, superoxide formation from peroxides and bicarbonate was confirmed in a reaction system with NaHCO_3_ and *m*CPBA. *m*CPBA is a peroxycarboxylic acid that is widely used as an oxidant in the laboratory. Peroxy acids, a major class of peroxides, can be substrates of peroxidase and are known to be present in CS [4,9]. As expected, the WST-1 signal increased by *m*CPBA and bicarbonate, and such increase was significantly attenuated by TEMPOL but not the hydroxyl radical scavenger mannitol (Figure 3B). Indeed, superoxide production from *m*CPBA and bicarbonate was reconfirmed with EPR analysis (Figure 4B, bottom panel). More than half of the CSE-generated superoxide appeared to originate from chemical reactions involving the peroxides.

### 3.4. The Species of Reactive Oxygen Was Reconfirmed to Be Superoxide

The species of reactive oxygen produced by CSE was examined with EPR spin-trapping experiments using a spin trap DIPPMPO [21,22]. A typical EPR spectrum for the superoxide spin adduct (DIPPMPO-OOH) was obtained from a xanthine/xanthine oxidase superoxide generating system (Figure 4A, first panel). Intriguingly, the EPR spectrum obtained from CSE in NaHCO_3_ solution was different from that of superoxide spin adduct (Figure 4B, first panel), as it overlapped with the spectrum of the hydroxyl radical spin adduct (DIPPMPO-OH) that was derived from the Fenton reaction between H_2_O_2_ and FeSO_4_ (Figure 4A, second panel). Nonetheless, the EPR signal from CSE was almost completely abolished by SOD (Figure 4B, second panel), whereas catalase and mannitol had little effect (Figure 4B, third and fourth panels, respectively). In short, CSE appeared to produce superoxide, but the signal obtained from EPR spectroscopy was that of hydroxyl radical spin adduct rather than superoxide spin adduct.

According to the previous observations, superoxide spin adduct can be reduced to hydroxyl radical spin adduct by reductants such as CBA [23,24]. Indeed, in the presence of CBA, the EPR spectrum obtained from the xanthine/xanthine oxidase superoxide generating system was that of hydroxyl radical spin adduct under this experimental system (Figure 4A, third panel). These results, together with those from previous studies, suggest that CSE generates superoxide, and the superoxide spin adduct is converted to hydroxyl radical spin adduct by certain constituents in CSE. This interpretation is additionally shown by the ineffectiveness of mannitol on superoxide formation detected with WST-1 (Figure 4C).

The pretreatment of CSE with HRP resulted in a significant decrease in the EPR signal (Figure 4B, fifth panel), which is consistent with the results obtained with WST-1 (Figure 3A).

### 3.5. TPM Also Generates Superoxide by Reacting with Bicarbonate

**Figure 5:**
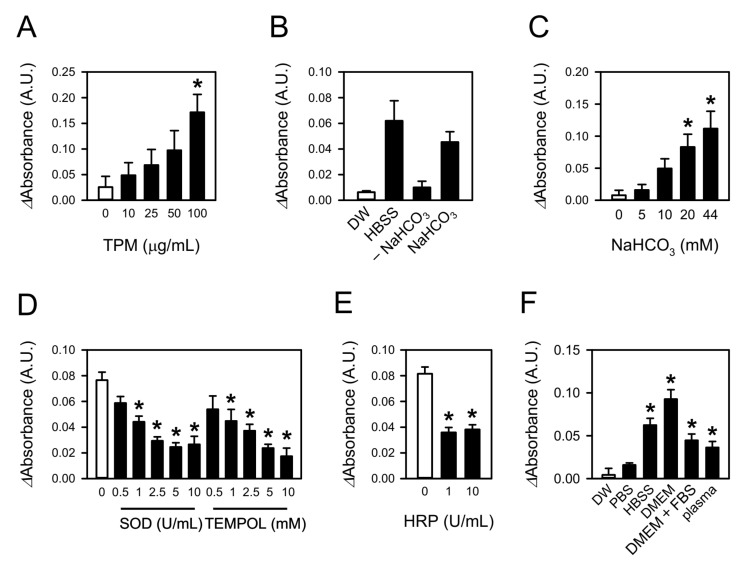
TPM also generates superoxide by reacting with bicarbonate. (**A**) Superoxide formation was measured in 44 mM NaHCO_3_ containing TPM using WST-1. (**B**,**C**) Superoxide production by 100 µg/mL TPM was assessed in DW, HBSS, HBSS deficient in NaHCO_3_ (–NaHCO_3_), and 4.16 mM NaHCO_3_ solution (NaHCO_3_) (**B**) or aqueous solutions with the indicated concentrations of NaHCO_3_ (**C**). (**D**) Absorbance of WST-1 formazan was measured in 100 µg/mL TPM-treated 44 mM NaHCO_3_ solution containing SOD or TEMPOL. (**E**) TPM was pretreated with HRP and added to 44 mM NaHCO_3_ solution. Superoxide formation was assessed with WST-1. (**F**) TPM-induced superoxide generation was measured in aqueous media, DW, PBS, HBSS, DMEM, DMEM containing 10% FBS, and blood plasma. Values are means ± standard errors (*n* = 5 for A, D, and F, *n* = 3 for B and E, and *n* = 4 for C). * *p* < 0.05 vs. corresponding control.

Superoxide formation by TPM was also examined. As with CSE, adding TPM produced superoxide in 44 mM NaHCO_3_ solution in a concentration-dependent manner over a range of 10−100 µg/mL (Figure 5A). Superoxide production was also observed in HBSS, and this formation was abolished by removing the NaHCO_3_ from HBSS (Figure 5B). The extent of superoxide formation in HBSS was comparable to that in a solution with the same concentration of NaHCO_3_ as HBSS. Superoxide formation by TPM was proportional to the concentration of NaHCO_3_, up to at least 44 mM (Figure 5C). The increase in the WST-1 signal by 100 µg/mL TPM in the 44 mM NaHCO_3_ solution was attenuated by 0.5−10 U/mL SOD and 0.5−10 mM TEMPOL (Figure 5D). The amount of superoxide generated was reduced by more than half by pretreating the TPM with HRP (Figure 5E). Similar to CSE, TPM only generated a significant amount of superoxide in the aqueous solutions containing NaHCO_3_ (Figure 5F).

### 3.6. CSE-Generated Superoxide Is Capable of Inducing Oxidative Modification of Albumin

**Figure 6:**
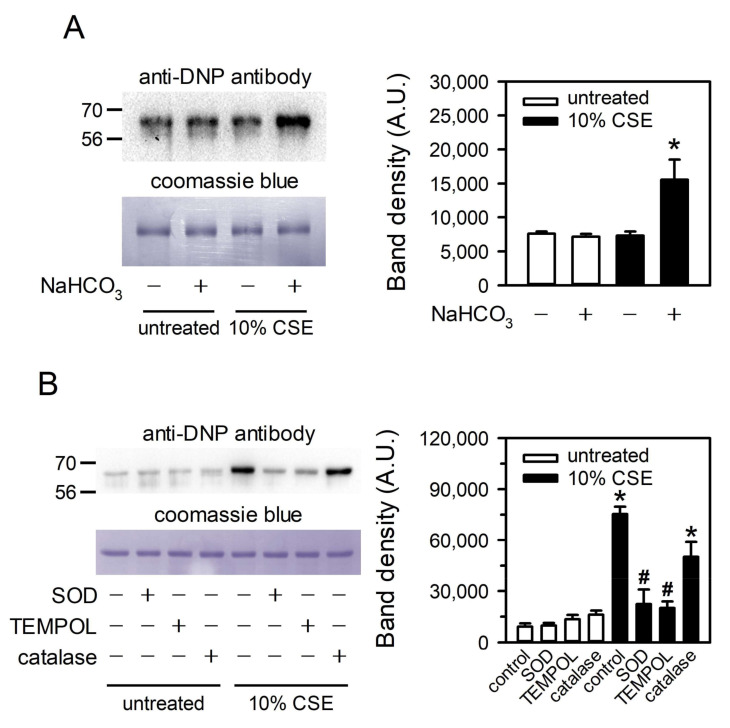
CSE-generated superoxide is capable of inducing oxidative modification of albumin. (**A**) Albumin was added to DMEM (+) or DMEM lacking NaHCO_3_ (–) to a concentration of 1 mg/mL. (**B**) DMEM containing 1 mg/mL albumin was treated with SOD, TEMPOL, or catalase. These albumin solutions were treated with 10% CSE for 5 min in the presence of 100 µM DTPA. Carbonylation of albumin was assessed using the Oxyblot protein oxidation detection kit. Coomassie blue staining is presented to show the amount of loaded albumin. Representative images and band intensities of carbonylated albumin are presented in the left and right panels, respectively. Values are means ± standard errors (*n* = 4). * *p* < 0.05 vs. CSE-untreated DMEM. ^#^ *p* < 0.05 vs. CSE-treated DMEM.

The biochemical impact of CSE-generated superoxide was tested with albumin, one of the most abundant proteins in body fluids. The oxidative modification of albumin was examined by immunoblot detection of the carbonyl groups introduced into the side chains of amino acids. CSE increased the carbonylation of albumin in DMEM, and this increase was not observed in DMEM lacking NaHCO_3_ (Figure 6A). In addition, CSE-induced albumin carbonylation was nearly completely prevented by SOD and TEMPOL, and partially prevented by catalase (Figure 6B), suggesting that the superoxide derived from CSE and NaHCO_3_ is capable of oxidizing macromolecules.

## 4. Discussion

CSE induces the generation of superoxide in cell culture systems not only by stimulating cells [8] but also through direct chemical reactions with culture media. Both CSE and TPM are able to produce superoxide in biocompatible aqueous media, such as HBSS, DMEM, and blood plasma (Figure 1A and Figure 5F). The major constituent contributing to superoxide formation in these media is bicarbonate (Figure 2 and Figure 5B). The substrates of peroxidase, such as peroxides, in CS preparations are responsible for more than half of the superoxide formation, presumably through chemical reactions with bicarbonate, although additional constituents must also be involved (Figure 3A and Figure 5E). Superoxide produced from CSE and bicarbonate has the potential to cause the oxidative modification of proteins (Figure 6). Our study suggests that in experimental conditions, the presence of bicarbonate should be considered carefully in in vitro studies of CS. In addition, the local amount or concentration of bicarbonate in exposed tissues might be a determinant of tissue sensitivity to oxidative damage by CS exposure.

CSE and TPM are the CS preparations most widely used for toxicological studies [25]. A large part of CS activity is attributed to excessive ROS formation and oxidative stress [1]; hence, the biological activities of CS are largely determined by its ROS-generating capability in diverse experimental systems. Media used for the culture or treatment of cells consist of a diverse complexity of components, and generally, their bicarbonate concentrations also vary from several millimoles to several tens of millimoles. Since CS-induced superoxide production is dependent on the amount of bicarbonate in the media, the biological activities of CS might be affected by the bicarbonate concentration in experimental systems. Indeed, superoxide arising from the chemical reaction between bicarbonate and CS was biologically active enough to induce the oxidative modification of macromolecules such as proteins (Figure 6). Therefore, the concentration of bicarbonate in the experimental system should be considered carefully in toxicity studies of CS and in the interpretation of experimental results.

The bicarbonate level is variable depending on the organ or tissue in body that is studied; for instance, the level is approximately 25 mM in serum and 14.4 mM in cytosol [26]. As is well known, bicarbonate levels can be altered by acid-base disorders [27]. In general, blood bicarbonate becomes elevated in respiratory alkalosis such as in Cushing’s syndrome or during the compensatory response to acidosis [28,29]. Local concentrations of bicarbonate may fluctuate more dynamically, and pulmonary alveolae may be more vulnerable to acid-base disturbances because they are a site of gas exchange [30]. The regulation of bicarbonate in the airways seems to be a complex process, and bicarbonate secretion mechanisms in airway epithelia have not been fully elucidated [31,32]. However, bicarbonate levels are related to mucus secretion and pathological conditions such as asthma and emphysema, thus, they reportedly increase in the late stage of chronic obstructive pulmonary diseases [33], and tissues may be more vulnerable to the oxidative damage by CS at this time. Intriguingly, interleukin (IL)-17A is known to stimulate bicarbonate secretion in bronchial epithelial cells [34], and CS-induced emphysema is milder in IL-17A-deficient than normal mice [35]. The findings of this study need to be considered when attempting to understand the pathogeneses of disorders caused by smoking and to define populations susceptible to CS toxicity.

CS is known to produce ROS in biological systems, but the molecular mechanisms underlying this ROS generation is not fully understood [11,36]. CS consists of numerous chemicals, including unidentified and uncharacterized constituents [1,3], and thus it was not possible to identify all the chemicals involved in superoxide production in our study. Representative processes responsible for ROS formation include redox cycling of quinone compounds and chemical reactions involving free iron or copper [9,36,37]. However, the substrates of peroxidase, i.e., peroxides, are responsible for more than half of the total superoxide production, at least under these experimental conditions (Figure 3A and Figure 5E) [19,20]. Nonetheless, there must be additional constituents involved in superoxide production through reactions with bicarbonate, considering that a substantial amount of the superoxide generation was not prevented by the pretreatment of CS with peroxidase, and superoxide production was completely abolished in the absence of bicarbonate. Clearly, further studies are needed to identify more of the constituents of CS.

Contrasting with our initial speculation, the EPR spectrum observed when CSE was added to NaHCO_3_ solution was not that of superoxide spin adduct but that of hydroxyl radical spin adduct. DIPPMPO forms a relatively stable superoxide adduct that does not convert into hydroxyl radical adduct without any further chemical reactions [38]. However, the EPR signal was completely abolished by SOD, whereas it was unaffected by catalase and mannitol (Figure 4B), suggesting that hydroxyl radical spin adduct is derived from superoxide. A possible mechanism underlying this observation is the rapid reduction of superoxide spin adduct to hydroxyl radical spin adduct by CSE. The EPR spectrum of hydroxyl radical spin adduct could be obtained from the xanthine/xanthine oxidase superoxide generating system in the presence of reducing agents such as boronate, glutathione, and ascorbic acid [23,38], and such a result was reproduced with CBA in this study (Figure 4A). Therefore, it is plausible that CSE contains reducing agents that act like CBA and thus can convert superoxide spin adduct to hydroxyl radical spin adduct. Catechols or phenolic compounds such as polyhydroxybenzenes are potential candidates for such reducing agents [39,40].

Superoxide production by CSE was greater in DMEM than HBSS, which is consistent with their bicarbonate concentrations of 44 mM and 4.16 mM, respectively. The bicarbonate concentration of rat plasma is approximately 25 mM [41], but the amount of superoxide produced in plasma did not exceed that in HBSS (Figure 1A and Figure 5F). This discrepancy may be attributed to the characteristics of plasma, which includes antioxidants such as albumin and vitamin E [42]. This interpretation is also supported by the observation that superoxide production in DMEM was reduced by the addition of FBS. One question remaining is how superoxide production was attenuated by removing NaCl from the HBSS (Figure 2C). Given that this attenuation was restored by compensating with NaF or KCl (Figure 2D), and NaCl is the predominant compound in HBSS (Section 2.5), ionic concentration appears to matter. Nevertheless, superoxide formation in NaHCO_3_ solution, which consists of only NaHCO_3_ and lacks NaCl, was comparable to that in HBSS (Figure 2A). We concluded the chemical reactions generating superoxide may be very complicated, and comprehensive studies will be required to further elucidate their characteristics.

WST-1 has been conventionally and widely used for the detection of superoxide [18]. However, contrary to the common recognition, we observed the increase of WST-1 signal by hydroxyl radical generated using H_2_O_2_ and NaHCO_3_, and its attenuation by mannitol (data not shown). This raises the possibility that WST-1 may detect the hydroxyl radical in addition to superoxide. The specificity of WST-1 toward superoxide needs to be explored although our current observation may not be enough to contradict the general notion that WST-1 is a superoxide probe.

## Data Availability

All study data are contained in the article.
